# Tuned activation of MSLN-CAR T cells induces superior antitumor responses in ovarian cancer models

**DOI:** 10.1136/jitc-2022-005691

**Published:** 2023-02-01

**Authors:** Esther Schoutrop, Thomas Poiret, Ibrahim El-Serafi, Ying Zhao, Rui He, Alina Moter, Johan Henriksson, Moustapha Hassan, Isabelle Magalhaes, Jonas Mattsson

**Affiliations:** 1 Department of Oncology-Pathology, Karolinska Institutet, Stockholm, Sweden; 2 Basic Medical Sciences Department, College of Medicine, Ajman University, Ajman, UAE; 3 Department of Biochemistry, Faculty of Medicine, Port-Said University, Port-Said, Egypt; 4 Experimental Cancer Medicine, Division of Clinical Research Center, Department of Laboratory Medicine, Karolinska Institutet, Stockholm, Sweden; 5 Clinical Research Center and Center of Allogeneic Stem Cell Transplantation (CAST), Karolinska University Hospital Huddinge, Stockholm, Sweden; 6 Laboratory for Molecular Infection Medicine Sweden (MIMS), Umeå Centre for Microbial Research (UCMR), Department of Molecular Biology, Umeå University, Umeå, Sweden; 7 Department of Clinical Immunology and Transfusion Medicine, Karolinska University Hospital, Stockholm, Sweden; 8 Gloria and Seymour Epstein Chair in Cell Therapy and Transplantation, Princess Margaret Cancer Centre and University of Toronto, Princess Margaret Cancer Centre, University Health Network, Toronto, Ontario, Canada

**Keywords:** Immunotherapy, Receptors, Chimeric Antigen

## Abstract

**Background:**

Limited persistence of functional CAR T cells in the immunosuppressive solid tumor microenvironment remains a major hurdle in the successful translation of CAR T cell therapy to treat solid tumors. Fine-tuning of CAR T cell activation by mutating CD3ζ chain immunoreceptor tyrosine-based activation motifs (ITAMs) in CD19-CAR T cells (containing the CD28 costimulatory domain) has proven to extend functional CAR T cell persistence in preclinical models of B cell malignancies.

**Methods:**

In this study, two conventional second-generation MSLN-CAR T cell constructs encoding for either a CD28 co-stimulatory (M28z) or 4-1BB costimulatory (MBBz) domain and a novel mesothelin (MSLN)-directed CAR T cell construct encoding for the CD28 costimulatory domain and CD3ζ chain containing a single ITAM (M1xx) were evaluated using in vitro and in vivo preclinical models of ovarian cancer. Two ovarian cancer cell lines and two orthotopic models of ovarian cancer in NSG mice were used: SKOV-3 cells inoculated through microsurgery in the ovary and to mimic a disseminated model of advanced ovarian cancer, OVCAR-4 cells injected intraperitoneally. MSLN-CAR T cell treatment efficacy was evaluated by survival analysis and the characterization and quantification of the different MSLN-CAR T cells were performed by flow cytometry, quantitative PCR and gene expression analysis.

**Results:**

M1xx CAR T cells elicited superior antitumor potency and persistence, as compared with the conventional second generation M28z and MBBz CAR T cells. Ex vivo M28z and MBBz CAR T cells displayed a more exhausted phenotype than M1xx CAR T cells as determined by co-expression of PD-1, LAG-3 and TIM-3. Furthermore, M1xx CAR T cells showed superior ex vivo IFNy, TNF and GzB production and were characterized by a self-renewal gene signature.

**Conclusions:**

Altogether, our study demonstrates the enhanced therapeutic potential of MSLN-CAR T cells expressing a mutated CD3ζ chain containing a single ITAM for the treatment of ovarian cancer. CAR T cells armored with calibrated activation potential may improve the clinical responses in solid tumors.

**WHAT IS ALREADY KNOWN ON THIS TOPIC**

- To date, chimeric antigen receptor (CAR) T cell therapy has not been very successful for the treatment of solid tumors.

**WHAT THIS STUDY ADDS**

- We compared in preclinical models mesothelin (MSLN) targeting CAR constructs for ovarian cancer treatment. Our study shows that T cells expressing the MSLN CAR construct that includes a CD3ζ chain containing a single immunoreceptor tyrosine-based activation motifs (M1xx), increased survival and M1xx CAR T cells displayed a less exhausted phenotype ex vivo.

**HOW THIS STUDY MIGHT AFFECT RESEARCH, PRACTICE OR POLICY**

- Calibration of CAR T cell activation is a promising strategy to enhance CAR T cell antitumor functions.

## Introduction

Since the first reported clinical application of CD19-directed chimeric antigen receptor (CAR) T cell therapy for the treatment of B cell malignancies major advances have been made in CAR therapy.^1 2^ To date, four different CD19-directed CAR constructs, containing either a CD28 or 4-1BB co-stimulatory domain, have been Food and Drug Administration (FDA) and European Medicines Agency (EMA) approved to treat CD19+ hematological malignancies.^3–6^

The mesothelin (MSLN) glycoprotein, overexpressed in many solid tumors, is a relevant target for antigen-specific therapies. Promising results have been reported using MSLN-directed CAR T cells in preclinical models^7 8^ and a recent phase I clinical trial demonstrated the safety profile of MSLN as a target antigen for CAR T cell therapy in patients with advanced solid cancers.^9 10^ Despite improvements in CAR design, successful clinical responses in solid tumors remain to be achieved. The solid tumor niche imposes several barriers: (1) physical barrier leading to poor immune trafficking and infiltration, (2) limited expression of tumor-specific/associated antigens and (3) immunosuppressive and hypoxic tumor microenvironment.^11 12^

One of the current challenges in CAR T cell therapy is to design a cell product capable of inducing potent and persistent antitumor responses in the immunosuppressive tumor microenvironment. Different strategies to boost CAR T cell antitumor efficacy have been explored: (1) CAR T cells expressing cytokine, chemokine or chemokine receptors^13 14^; (2) engineered downregulation of coinhibitory molecules (CIMs) such as PD-1, TIM-3, and LAG-3^15 16^; (3) combinational therapies with immune checkpoint blockade^17^ or oncolytic viruses.^18 19^ Finetuning of intracellular CAR signaling, through mutation of the two distal immunoreceptor tyrosine-based activation motifs (ITAMs) of the CD3ζ chain, has proven to be a promising approach to increase CD19-CAR T cells potency.^20–22^

We compared the potential therapeutic effect of M1xx, a MSLN-directed CAR T cell containing a CD28 costimulatory domain and a CD3ζ chain with mutations in the two distal ITAMs, with two conventional second-generation MSLN-CAR T cells containing either CD28 (M28z) or 4-1BB (MBBz) costimulatory domain.^20^ Functionality and therapeutic efficacy of M1xx CAR T cells was assessed in vitro and in two in vivo orthotopic preclinical models of ovarian cancer.

## Material and methods

### CAR T-cell production

**Figure 1:**
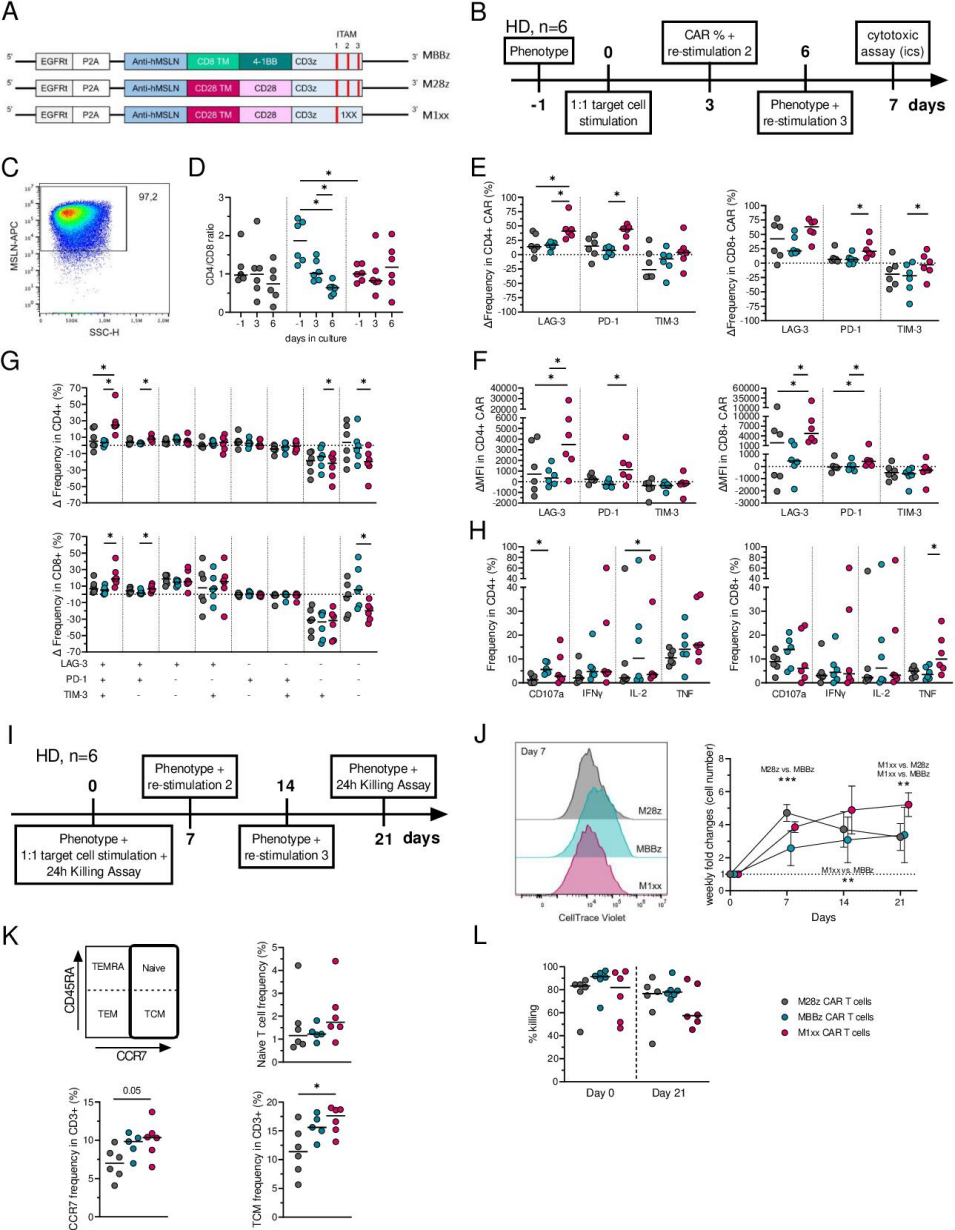
Impact of repeated In vitro stimulation on MSLN-CAR T cells. A. MSLN-directed constructs with a CD3ζ endodomain and 4-1BB costimulatory domain (MBBz) or CD28 costimulatory domain (M28z) or CD28 with ITAM2-ITAM3 mutated CD3ζ chain (M1xx). (B) Experimental design short-term restimulations assay, number of healthy donors, n=6. (C) Representative plot of MSLN expression in K562 target cells. (D). Kinetics of the CD4^+^/CD8^+^ ratio within the different MSLN-CAR T cells. (E–F) LAG-3, PD-1 and Tim-3 CIM expression kinetics by MSLN-CAR T cells prior to and following antigenic (re)stimulation, as determined by frequency (E) and MFI (F, G) changes in CIM coexpression by CD4^+^ (top) and CD8^+^ (bottom) MSLN-CAR T cells prior to and following antigenic (re)stimulation. (H) frequency of CD107a, IFNγ, IL-2 and TNF producing CD4^+^ (left) and CD8^+^ (right) T cells after three stimulations with K562 MLSN+target cells. (I) Experimental design of the long-term restimulations assay spanning 3 weeks. Number of healthy donors: n=6. (J) Representative histogram of proliferation as determined by CellTrace violet signal by the different MSLN-CAR T cell constructs at day 7 post-first stimulation (left) and weekly monitoring (cell count) of MSLN-CAR T cell proliferation (right). (K) Expression of CD45RA and CCR7 at day 21 (following 3-week period in vitro stimulation). (L) Percentage killing of K562 MSLN^+^GFP^+^ target cells by the different MSLN-CAR T cells after a single exposure (day 0, left) and three exposures (day 21, right, means and SD are represented). The two-way ANOVA with Sidak’s test was used to do multiple comparisons between CAR T cells and date. Friedman’s test with Dunn’s correction was used to compare. The three paired MSLN-CAR T cells. delta Δ=value at day 6 – value at day 0. Medians are represented. *p<0.05, **p<0.01,***p<0.001. ANOVA, analysis of variance.

Peripheral blood mononuclear cells were isolated from healthy donor buffy coats (Karolinska University Hospital, Huddinge, Sweden) using Ficoll-Paque density gradient centrifugation (GE Healthcare). T cell activation and viral transduction with γ-retroviral vectors encoding for three distinct MSLN-directed CAR constructs was performed as reported previously.^23^ The MSLN-directed CAR constructs (generously provided by Prof. M. Sadelain, Memorial Sloan Kettering Cancer Center (MSKCC), New York, USA), encompass a human MSLN-specific ScFv,^21^ followed by either a CD28-CD3ζ, 4–1BB-CD3ζ or a CD28 costimulatory domain linked to an ITAM2-ITAM3 mutated CD3ζ chain. The CAR constructs were linked by 2A self-cleaving peptide to a truncated EGFR (EGFRt) sequence, allowing assessment of transduction efficiency (figure 1A). Anti-human CD19-CAR construct containing the CD28-CD3ζ signaling domain was included as control (kindly gifted by Prof. S. Rosenberg, NCI, Bethesda, USA). Retroviral supernatants were manufactured as previously reported.^23–25^

### Human cancer cell lines

The high-grade serous human ovarian adenocarcinoma cell line, OVCAR-4, was generously provided by Prof. Kaisa Lehti (Karolinska Institutet, Stockholm, Sweden) and cultured in RPMI-1640 (Hyclone). SKOV-3 tumor cells (human ovarian adenocarcinoma ATCC, HTB-77) were cultured in McCoy’s 5a medium (Sigma-Aldrich). The chronic myelogenous leukemia cell line, K562, was maintained in RPMI-1640. All culture media were supplemented with 10% fetal bovine serum (FBS, GE Healthcare) and 1% penicillin-streptomycin (Gibco). Tumor cells were transduced with γ-retroviral vectors encoding for human MSLN variant 1 (SFG vector) and green fluorescent protein (GFP)/firefly luciferase fusion protein (SFG vector) (kindly donated by Prof. M. Sadelain, MSKCC). To isolate a pure polyclonal pool of double-positive MSLN^+^GFP^+^/ffLuc^+^ cells, transduced OVCAR-4, SKOV-3 and K562 cells were sorted using Fluorescence-Activated Cell Sorting (FACS) (BD FACSAria, Becton Dickinson and SONY MA900 cell sorter, Sony Biotechnology). MSLN^+^GFP^+^/ffLuc^+^ OVCAR-4 and SKOV-3 cells were assessed for microbiology and Mycoplasma (MycoAlert Mycoplasma Detection Kit, Lonza) prior to injection in mouse models.

### Short-term in vitro restimulations assay

The phenotype of M28z, MBBz and M1xx CAR T cells was determined by flow cytometry prior to start of assays and on day 6 following two rounds of antigen stimulation. MSLN-CAR T cells were cocultured with irradiated (55 Gy) MSLN^+^ K562 cells at a 1:1 effector:target (E:T) ratio on day 0 (figure 1B). After 72 hours of coculture, CAR frequency was evaluated and MSLN-CAR T cells were restimulated with freshly irradiated MSLN^+^ K562 cells and cocultured at 1:1 E:T ratio for another 72 hours. On day 6, MSLN-CAR T cells were exposed for a third time to MSLN^+^ K562 cells at 1:1 E:T ratio and following 6 hours of coculture, cytokine production and degranulation were assessed by intracellular staining as previously described.^26^ Cocultures were maintained for a total of 6 days in AIM-V medium supplemented with 5% human AB serum, at 37°C, 5% CO_2_.

### Long-term in vitro restimulations assay

MSLN-CAR T cells were labeled with CellTrace Violet Cell Proliferation Kit (ThermoFisher Scientific) on day 0. CAR T cells phenotype was determined (flow cytometry) on days 0, 7, 14 and 2, stimulation done with irradiated (55 Gy) MSLN^+^ K562 cells, 1:1 E:T ratio (figure 1I). Manual cell counts and medium medium refreshments (AIM-V medium supplemented with 5% human AB serum and 300 IU IL-2) were performed on a weekly basis. Cells were incubated at 37°C, 5% CO_2_.

K562 MSLN^+^GFP^+^ cells were used to assess killing by MSLN-CAR T cells (day 0 and 21). Briefly, after CAR frequency evaluation (FACS), 0.1E6 MSLN-CAR T cells were incubated for 24 hours at 37°C, 5% CO_2_ in a 96-well plate with 0.2E6 K562 MSLN^+^GFP^+^ at a 1:2 E:T ratio. Donor-matched untransduced T cells were used as negative control. The assay was performed in a final volume of 200 µL AIM-V medium supplemented with 5% human AB serum. After 24 hours, 100 µL from each resuspended co-culture was added to 40 µL of ONE-Glo EX Reagent (Promega) and bioluminescence reading done using CLARIOstar multireader (BMG Labtech). MSLN- CAR T cells specific killing was calculated as follows:

$$\%specifickilling=100x⟮1-\frac{CARTcellsbiolum}{UntransducedTcellsbiolum}⟯$$

### SKOV-3 and OVCAR-4 mice models

Female NOD *scid* gamma (NSG) mice (Charles River) aged 6–8 weeks old were kept as described previously.^27^ An orthotopic model of ovarian cancer was established through microsurgery: 0.5E6 SKOV-3 MSLN^+^GFP^+^/ffLuc^+^ cells (passage 36 and 37) were injected into the bursa of the left ovary as reported previously.^27^ To mimic a disseminated model of ovarian cancer, 1E6 OVCAR-4 MSLN^+^GFP^+^/ffLuc^+^ cells (passage 28) were administered intraperitoneally (i.p.). Mice were treated with 1E6 CAR T cells (corresponding approximately to 1.5E6 to 2E6 total T cells) by intravenous injection 21–22 days post SKOV-3 tumor inoculation or i.p. injection 13 days after OVCAR-4 tumor engraftment, respectively. Prior to CAR T cell administration, mice were divided into treatment groups with comparable median bioluminescence imaging (BLI) signal intensity (used to monitor tumor burden).

Overall condition, body weight (online supplemental figure 2B, online supplemental figure 4C) and tumor burden were evaluated weekly. Mice were i.p injected with 150 mg/kg d-luciferin (PerkinElmer) in PBS to detect the ffLuc^+^ bioluminescence signal using the In Vivo Imaging System (IVIS, PerkinElmer) and data were analyzed with the Living Image V.4.3.1 software (PerkinElmer). BLI threshold detection level was evaluated at 1.5E6 photons using two untreated NSG mice. Some IVIS points were excluded due to technical error during BLI measurements.

10.1136/jitc-2022-005691.supp2 Supplementary data

10.1136/jitc-2022-005691.supp4 Supplementary data

To longitudinally characterize tumors and CAR T cells, some mice were sacrificed at intermediate time points (study design, online supplemental table 1). Remaining mice were kept for survival analysis and sacrificed on reaching humane endpoint as described previously.^27^

10.1136/jitc-2022-005691.supp5 Supplementary data

### Organ collection and processing

Primary ovarian tumor/ovarian tissue, spleens, blood (by heart puncture) and, if available, ascites were harvested on sacrificing of mice that received SKOV-3 cells. Organs were processed into single cell suspensions for flow cytometry and cell culture in case of sufficient material, as described previously.^27^

Spleens of sacrificed mice inoculated with OVCAR-4 cells were processed into single cell suspensions for flow cytometry analysis and FACS sorting (BD FACSAria, Becton Dickinson and SONY MA900 cell sorter, Sony Biotechnology) to isolate CAR-transduced T cells (CD45^+^CD3^+^EGFRt^+^). In parallel, the infused CAR fraction of M28z and M1xx CAR T cells (OVCAR-4 model) were also FACS sorted. Following sorting, isolated CAR^+^ T cells were counted and pelleted, dry cell pellets were stored at −80°C until further usage.

### Quantitative real-time PCR

Peripheral blood was collected from tail veins of SKOV-3-inoculated mice every 2 weeks. After centrifugation, blood pellets were stored at –80°C until usage. For DNA extraction, blood pellets from several mice (up to four) were pooled when needed to achieve an initial volume of collected blood between 60 and 200 µL. DNA extraction was performed using Qiagen AllPrep DNA/RNA kits (Qiagen). To detect MSLN-CAR T cells, primers specific for EGFRt (forward primer 5’-AATGCGTGGACAAGTGCAAC-3', reverse primer 5’-CGATGGACGGGATCTTAGGC-3') were used. PowerUP SYBR green master mix (Applied Biosystems) was mixed with primers and 25 ng DNA per reaction in Microamp Fast Optical 96-well reaction plates (Thermo Scientific), amplification was performed using a 7500 fast real-time PCR machine (Applied Biosystems). Primers specific for the beta-globulin gene (HBG) were included as internal control.

### Flow cytometry

Staining with biotinylated anti-EGFRt/Cetuximab antibody (R&D systems) was used to assess EGFRt expression and thus CAR transduction efficiency. Detection of CAR surface expression was using biotinylated anti-human Fab (for MSLN-CAR) or anti-mouse Fab (for CD19-CAR) antibodies followed by a blocking step with mouse γ-globulin (all from Jackson ImmunoResearch Lab). Stainings with anti-Fab or anti-EGFRt were followed by staining with streptavidin-PE conjugated antibody (Biolegend) (online supplemental figure 2A). Extracellular antibodies were included in the secondary staining (online supplemental tables 2 and 3). Cell viability was assessed with 7AAD. Tumor cells were checked regularly by flow cytometry for MSLN cell surface expression and GFP signal. Acquisition was performed on CytoFlex (Beckman Coulter) and data analyzed using FlowJo V.10.8 Software (BD Life Sciences).

10.1136/jitc-2022-005691.supp6 Supplementary data

10.1136/jitc-2022-005691.supp7 Supplementary data

### Fluorospot assays

MSLN-directed CAR T cells isolated from primary ovarian tumors and/or spleen of SKOV-3 MSLN^+^GFP^+^/ffLuc^+^ inoculated mice were cocultured with MSLN^+^ OVCAR-3 cells for 24 hours in FluoroSpot plates (FluoroSpotPLUS kit IFNy/GranzymeB/TNFa, Mabtech) allowing detection of IFNy, GzB and TNF production and analyzed using the IRIS^TM^ ELISpot/FluoroSpot reader (Mabtech).

### Gene expression analysis

Sorted CAR T cell pellets were thawed and resuspended in 1:3 diluted RLT buffer. Cell pellets of some mice (from the same treatment group) were pooled for Nanostring analysis (online supplemental table 4). Cells were then incubated for 20 min at 4°C for lysis. Lysed samples equivalent to 10,000 cells were analyzed for gene expression using the nCounter CAR-T Characterization and the nCounter Metabolic Pathways Panels (Nanostring Technologies) using the Nanostring MAX/FLEX platform (KIGene core facility, Karolinska Institutet, Stockholm, Sweden). nCounter data were analyzed with a custom script in R. Because 212 genes overlapped between the nCounter CAR-T Characterization and the nCounter Metabolic Pathway panels, the reproducibility of the measurements could be calculated. A linear model was set up over Log_2_ normalized nCounter measurements to remove any batch variations by a constant shift. The remaining difference between repeated measurements was assumed to be the real dispersion. The dispersion for all the genes was then estimated using the Bayesian approach of DEseq2, assuming that the error was normally distributed.^28^ Fold changes were calculated by subtraction in log space, with deviations calculated from the normal distribution using the square additivity of variances. The background value was set as negative controls maximum value +30 and was used for gene exclusion. List of the genes used for comparison analysis can be found in online supplemental table 5A-C.

10.1136/jitc-2022-005691.supp8 Supplementary data

10.1136/jitc-2022-005691.supp9 Supplementary data

### Data and statistical analysis

Data analysis was conducted using GraphPad Prism (GraphPad Software, San Diego, California, USA). Data are represented as median, unless indicated intermediate time points were pooled with humane endpoint data. Individual mice were used as experimental units, with a minimum of five mice per group for statistical analysis. Non-parametric paired samples of two or multiple groups were compared using Wilcoxon matched-pairs signed rank test or Friedman test with Dunn’s correction. Two groups and multiple groups of unpaired samples were compared using Mann-Whitney test or Kruskal-Wallis test, respectively. The two-way analysis of variance with Sidak’s test was used to do multiple comparisons between groups of samples overtime. Log-rank test was used to analyze Kaplan-Meier long-term survival curves. Correlation was analyzed by linear regression; log 10 transformation of the data was performed when necessary for correlation analysis. The threshold of significance was set at 0.05.

## Results

### MSLN-CAR T cell phenotype and function were impacted differently on repeated stimulations

Repeated in vitro stimulations of MSLN-CAR T cells were performed over a period of 1 week (short term restimulations assays) to compare the phenotype and functionality of M28z, MBBz and M1xx CAR constructs. The frequency of CAR^+^ T cells (online supplemental figure 1A) remained stable overtime for M28z and MBBz CAR T cells, while it decreased significantly at day 6 (after two stimulations) in M1xx CAR T cells. After two stimulations with MSLN^+^ K562 target cells (figure 1C), there was a shift towards a lower CD4^+^/CD8^+^ ratio within MBBz CAR^+^ T cells (figure 1D). Notably, MBBz CAR T cells exhibited a significantly higher CD4^+^/CD8^+^ ratio than M1xx CAR T cells prior to antigen exposure (figure 1D, online supplemental figure 1B). CIM expression levels were impacted differently following repeated stimulations with MSLN^+^ target cells on M28z, MBBz and M1xx CAR T cells. Following repeated antigen exposure, LAG-3 and PD-1 expression (as determined by frequency and median fluorescence intensity, MFI) increased significantly in MSLN-CAR T cells, especially in M1xx CAR T cells compared with M28z and MBBz CAR T cells (p<0.05, (online supplemental figure 1C–F, figure 1E, F). Accordingly, M1xx CAR T cells showed a stronger increase in phenotypically exhausted LAG-3/PD-1/TIM-3 triple positive CAR T cells than M28z (CD4^+^ only) and MBBz (both CD4^+^ and CD8^+^) CAR T cells (p<0.05) after repeated antigen exposure. The activation/exhaustion phenotype of MBBz CAR T cells was less impacted by repeated antigen stimulations than M1xx CAR T cells, as shown by the stable frequency of LAG-3/PD-1/TIM-3 triple negative cells within MBBz CAR T cells (p<0.05, figure 1G, online supplemental figure 1G, H).

10.1136/jitc-2022-005691.supp1 Supplementary data

Following the third antigen exposure, M28z CD4^+^ CAR T cells showed less degranulation (CD107a^+^) than MBBz CAR T cells and produced less IL-2 than M1xx CAR T cells, while CD8^+^ M1xx CAR T cells were more efficient in TNF production than MBBz CAR T cells (p<0.05, figure 1H). There were no differences between MSLN-CAR T cells concerning their multifunctionality, and noticeably MSLN-CAR T cells demonstrated mainly one or two functions on repeated antigen exposure (online supplemental figure 1I, J).

Repeated in vitro stimulations were then performed over a 3-week period (long-term restimulations assay) including weekly stimulations with MSLN^+^ K562 target cells (figure 1I). Overall, CAR frequency within CD3+ cells increased progressively up to a median >90% (ie, M28z 95,9%, MBBz 92,1% and M1x×98,5%) while the CD4/CD8 ratio progressively decreased (p<0.001, online supplemental figure 1K). While M28z CAR T cells displayed a rapid initial proliferation capacity during the first week of antigenic exposure (p<0.001 vs. MBBz CAR T cells), M1xx CAR T cells demonstrated a more persistent expansion potential (as measured by proliferation and cell counts) over 21 days with repeated antigen exposure (p<0.01 vs. M28z and MBBz CAR T cells, figure 1J). At 21 days, M1xx CAR T cells presented a higher frequency of LAG-3 and TIM-3 surface expression as compared with M28z and MBBz CAR T cells (p<0.01). No differences in PD-1 expression levels were observed between the MSLN-CAR T cell constructs (online supplemental figure 1L). M1xx CAR T cells exhibited the highest CCR7 expression levels among the MSLN-CAR T cells (p=0.05) which translated into a higher central memory phenotype (TCM, p<0.05) compartment as compared with M28z CAR T cells (figure 1K). Overall, no differences in killing potential between MSLN-CAR T cells after a single (day 0) or after repeated (day 21) exposure to target cells were detected (figure 1L).

### M1xx CAR T cells induced persistent tumor regression and long-term remission in vivo

**Figure 2:**
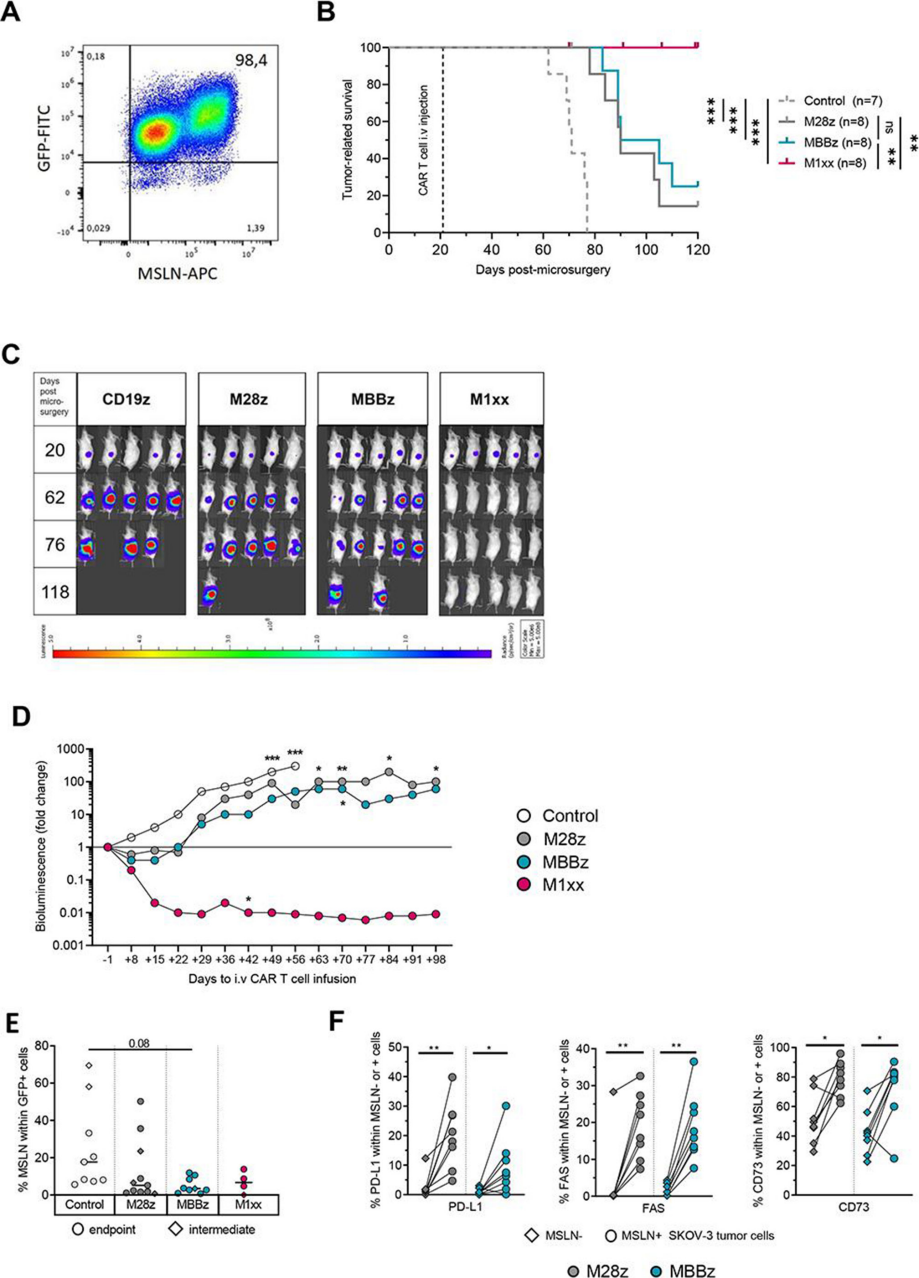
M1xx CAR T cells display superior tumor control against orthotopic SKOV-3 tumors in vivo. (A) Representative plot of MSLN expression in SKOV-3 ovarian cancer cells. (B) Kaplan-Meier curve representing the survival of control mice (n=7, CD19-CAR T cell treatment) and mice treated with MSLN-directed CAR T cells, containing distinct intracellular signaling domains: M28z (n=8), MBBz (n=8) or M1xx (n=8). (C) Overall representative weekly bioluminescence monitoring of the SKOV-3 tumor burden of NSG mice. (D) Comparison of the relative tumor growth (determined by BLI) between control mice and MSLN-CAR T cell-treated mice (control vs M28z vs MBBz vs M1xx, median represented) from the day prior to CAR treatment. (E) MSLN cell surface expression on GFP^+^ SKOV-3 tumor cells exposed to control or MSLN-CAR T cell treatment. (F) Expression levels of PD-L1, Fas and CD73 on MSLN^+^ and MSLN^−^ SKOV-3 tumor cells in M28z-treated and MBBz-treated mice. Log-rank test was used to compare the survival between groups of mice. The two-way ANOVA with Sidak’s test was used to do multiple comparisons between CAR T cells and date. Kruskal-Wallis test was used to compare the MSLN expression in SKOV-3 cells of different groups of mice. Wilcoxon matched-pairs signed RANK test was used to compare marker expression between MSLN^+^ and MSLN^−^ SKOV-3 cells within same mice. Medians are represented. *p<0.05, **p<0.01, ***p<0.001 ANOVA, analysis of variance.

To assess the antitumor efficacy of M28z, MBBz and M1xx CAR T cells in an in vivo model, NSG mice were inoculated with MSLN^+^GFP^+^/ffLuc^+^ SKOV-3 cells (figure 2A), followed by M28z, MBBz or M1xx CAR T cell intravenous treatment 21 days post tumor engraftment. Treatment with all MSLN-CAR constructs significantly prolonged survival compared with control mice (figure 2B, C, online supplemental figure 2C). Median BLI signals already decreased 1 week after MSLN-CAR treatment compared with the control group (p<0.001), however, only M1xx CAR T cells induced persistent tumor regression (figure 2D). Indeed, relative to BLI prior to CAR treatment (−1 day), median BLI signal decreased up to 100-fold and below level of detection (online supplemental figure 2C) in some mice treated with M1xx CAR T cells. Treatment with M28z and MBBz CAR T cells merely delayed tumor progression with median BLI signals reaching the initial BLI level (day –1) within 29 days. Accordingly, tumor burden was significantly reduced in M1xx CAR T cell treated mice compared with both the M28z-treated and MBBz-treated groups over time (p<0.05). The remarkable antitumor effect elicited by M1xx CAR T cells was maintained until termination of the experiment (>90 days post CAR treatment), with BLI signals on the threshold of detection, demonstrating long-term remission (figure 2D).

### Loss of MSLN surface expression and PD-L1, FAS and CD73 upregulation by SKOV-3 tumor cells

SKOV-3 tumor cells were detected in all primary tumors isolated from the control, M28z and MBBz treatment groups, but only detectable in 4/10 mice treated with M1xx CAR T cells. At time of microsurgery, MSLN cell surface expression by SKOV-3 cells was 94% (median of six independent samples). Regardless of treatment, median MSLN surface expression decreased below 20% at the time of sacrifice. Of note, in the control group high frequency of MSLN^+^ tumor cells was detected at the intermediate time point and lower at the humane endpoint, while the frequency of MSLN^+^ tumor cells in MSLN-CAR-treated mice was low at both the intermediate and humane endpoint (figure 2E). Linear regression analysis of tumor weight on sacrifice and MSLN expression by tumor cells revealed a negative correlation within the control treatment group. No correlations were found in MSLN CAR T cell-treated groups (online supplemental figure 2D). This suggests that MSLN surface expression in control mice gradually decreased during tumor progression, whereas MSLN-CAR therapy accelerated the loss of MSLN surface expression. PD-L1^+^, FAS^+^ and CD73^+^ tumor cells were found at higher frequencies within MSLN^+^ SKOV-3 cells compared with MSLN^-^ SKOV-3 cells, regardless of treatment group M28z and MBBz (figure 2F). Ex vivo phenotypic analysis of SKOV-3 tumor cells was not possible in M1xx-treated mice due to their absence or low frequency in primary tumor/ovarian tissues.

### SKOV-3 tumor-derived and spleen-derived M1xx CAR T cells displayed superior functionality

**Figure 3:**
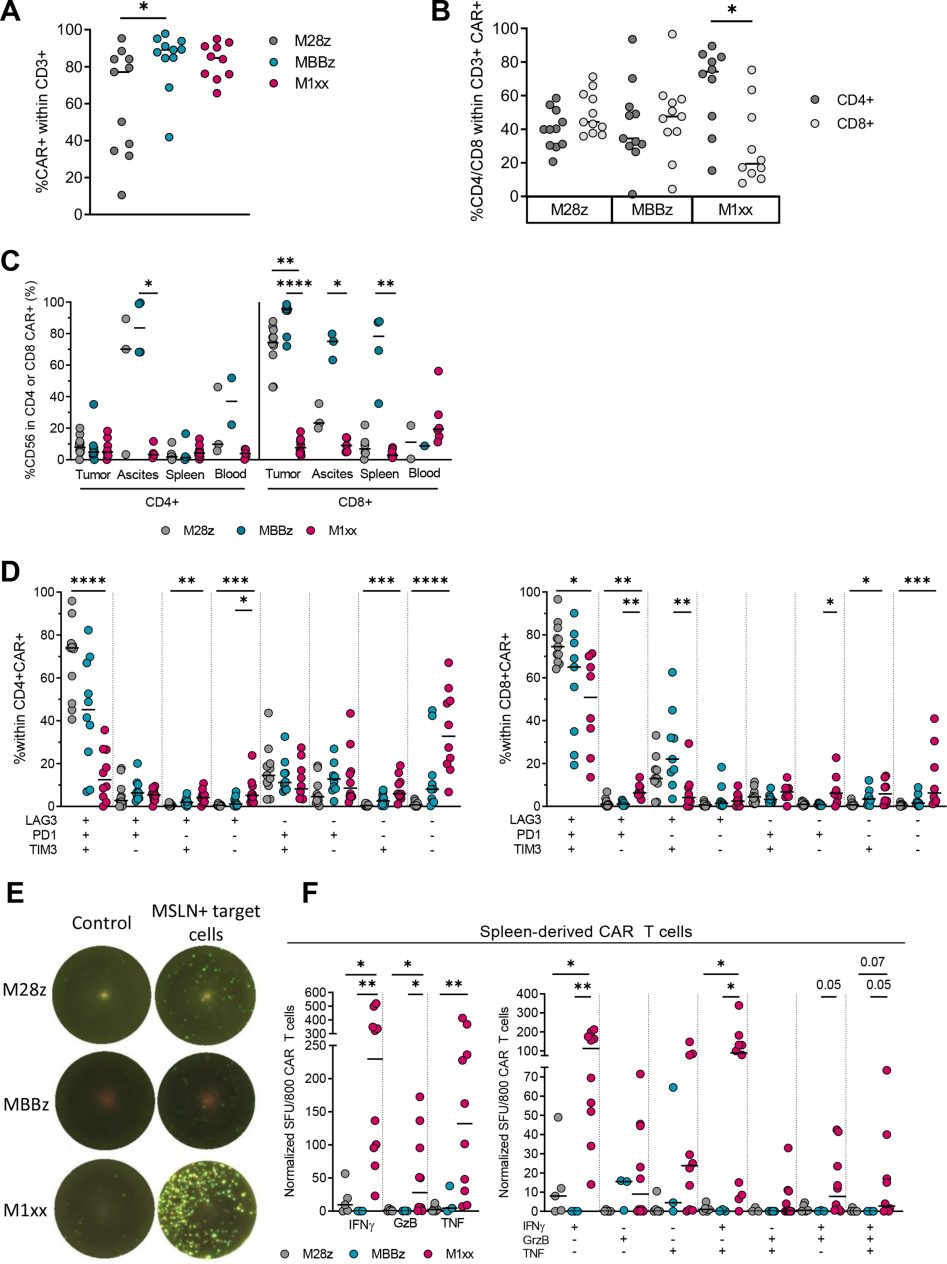
Ex vivo characterization of MSLN-CAR T cells isolated from SKOV-3 engrafted mice. (A) Comparison of CAR^+^ T cell fractions isolated from primary ovarian tumor/tissue of M28z-treated, MBBz-treated or M1xx-treated mice. (B) Comparison of CD4^+^ and CD8^+^ frequency within CAR^+^ T cells found within primary ovarian tumor/tissue. (C) CD56 expression in M28z, MBBz and M1xx CAR T cells isolated from primary tumor, ascites, spleen or blood. (D) CIM coexpression pattern on CD4^+^ (left) and CD8^+^ (right) M28z, MBBz and M1xx CAR^+^ T cells derived from primary ovarian tumor/tissue. (E.) Representative Fluorospot images, displaying IFNy (green), GzB (yellow) and TNF (pink) (co)production by unstimulated (control) and antigen stimulated ex vivo MSLN-CAR T cells. (F) Number of IFNy, GzB and TNF spots forming unit (SFU) per 800 CAR T cells isolated from spleen of M28z-treated, MBBz-treated and M1xx-treated mice stimulated with MSLN^+^ target cells at a 1:1 ratio for 24 hours. Normalized SFU displayed following background subtraction with unstimulated spleen-derived MSLN-CAR T cells. Kruskal-Wallis test was used to compare the antigen expression between the three different groups of mice. Mann-Whitney test was used to compare CAR frequency between CD4^+^ and CD8^+^ T cells. Medians are represented. *p<0.05, **p<0.01, ***p<0.001, ****p<0.0001.

T cells were detectable in all primary tumor/ovarian tissues isolated from sacrificed mice, regardless of MSLN-CAR treatment group. Most tumor-infiltrating T cells were CAR-transduced; however, the median frequency of CAR-transduced T cells was significantly lower in the M28z than MBBz CAR treatment group (p<0.05, figure 3A). Interestingly, while CD4^+^ and CD8^+^ T cell frequencies were comparable within tumor-infiltrating M28z and MBBz CAR T cells, the CD4^+^ subset was predominant within M1xx CAR T cells (p<0.05, figure 3B).

At sacrifice, 70% of the M1xx-treated mice showed detectable presence of CAR T cells in blood samples by flow cytometry (>20 cells) as compared with 36% and 45% of M28z-treated and MBBz-treated mice, respectively (online supplemental figure 3A). These findings were confirmed by qPCR analysis, as CAR T cells were only detected in peripheral blood of M1xx CAR T cell-treated mice (online supplemental figure 3B).

10.1136/jitc-2022-005691.supp3 Supplementary data

In some mice, when detectable, we characterized MSLN-CAR T cells in ascites, spleen and blood. Overall, the frequency of CD56 was low on tumor-derived and spleen-derived CD4^+^ MSLN-CAR T cells. Notably, in ascites, the CD56 of frequency was lower in M1xx CD4^+^ CAR T cells (p<0.05) and CD8^+^ tumor-infiltrating M1xx CAR T cells (p<0.01), whereas high M28z and MBBz CAR T cells. The frequency of CD56^+^CD8^+^ cells was high within ascites-derived and spleen-derived MBBz CAR T cells compared with M28z and M1xx CAR T cells (figure 3C).

The frequency of LAG-3, PD-1 and TIM-3 CIM was substantially lower in CD4^+^ M1xx than CD4^+^ M28z CAR T cells. A similar trend was observed for MBBz CAR T cells relative to M1xx CAR T cells (online supplemental figure 3C). Furthermore, LAG-3 and TIM-3 frequencies were higher within CD8^+^ M28z and MBBz compared with M1xx CAR T cells, while PD-1 expression was comparable between the CD8^+^ MSLN-CAR T cells. Coexpression analysis revealed that the frequency of triple negative cells was elevated in M1xx CAR T cells, conversely increased levels of LAG-3/PD-1/TIM-3 triple positive cells were observed in M28z CAR T cells relative to M1xx CAR T cells (figure 3D). A similar trend was observed between MBBz and M1xx CAR T cells. Frequency of LAG-3, PD-1 and TIM-3 single positive cells was elevated within M1xx CAR T cells compared with M28z and MBBz. FASL frequency was significantly higher in M28z and MBBz CAR T cells relative to M1xx CAR T cells. Interestingly, the LAG-3, PD-1, TIM-3 and FASL expression profile on MSLN-CAR T cells was tissue-specific. Details can be found in online supplemental figure 3D.

Because of the higher abundance of MSLN-CAR T cells in the spleen (compared with other tissues that is, blood), spleen-derived MSLN-CAR T cells were rested overnight prior to co-culture with MSLN+target cells at a 1:1 effector to target ratio in FluoroSpot plates (figure 3E). Following coculture, M1xx CAR T displayed superior IFNy, GzB and TNF production compared with M28z and MBBz CAR T cells and were able to produce two to three soluble factors simultaneously, deeming them polyfunctional (figure 3F).

### Intraperitoneal M1XX CAR T cell treatment improved survival of mice with peritoneal disseminated ovarian cancer

**Figure 4:**
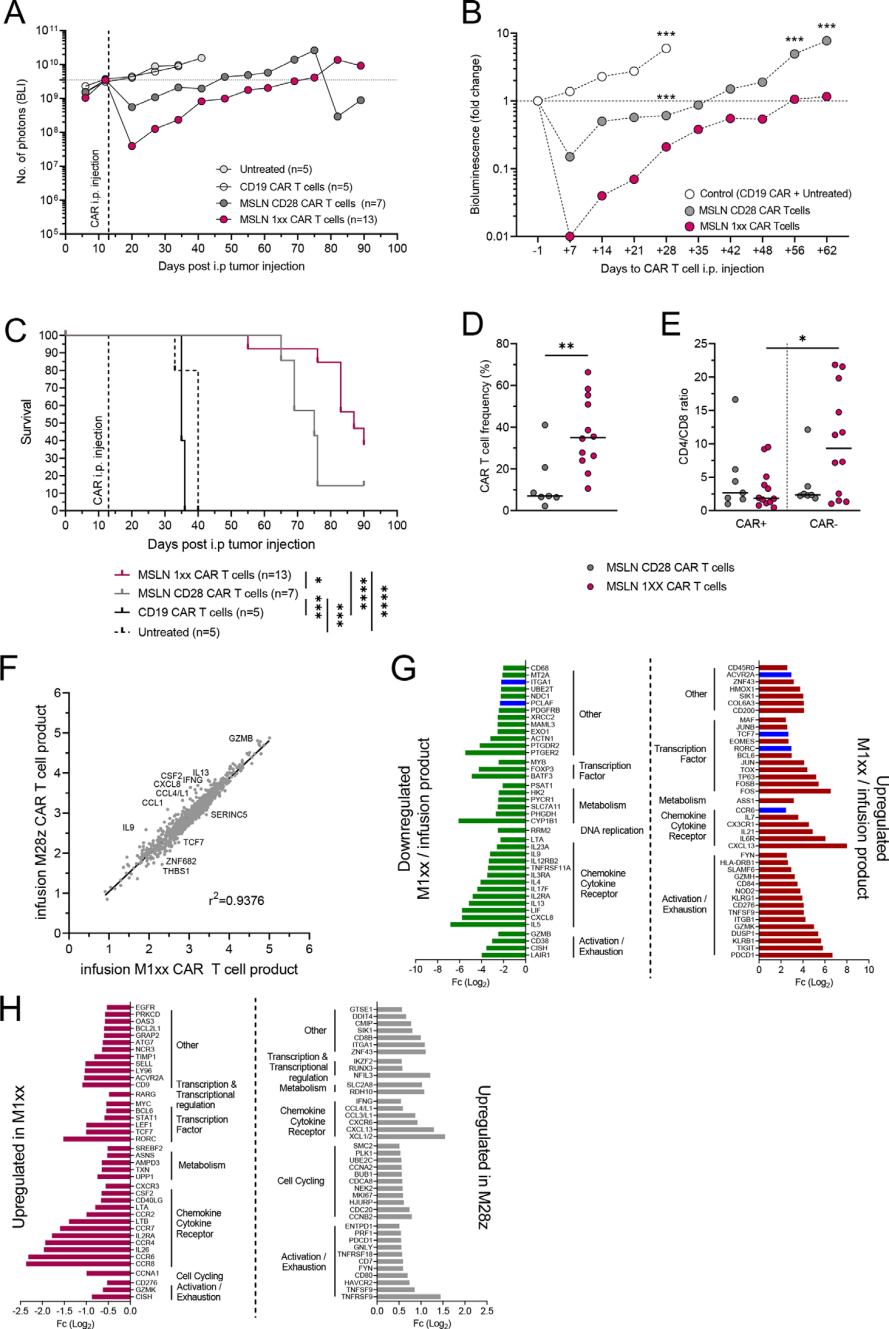
MSLN 1 xx CAR T cell improved survival against peritoneal disseminated ovarian cancer cells. (A) Weekly monitoring of tumor burden in NSG mice intraperitoneally inoculated with OVCAR-4 MSLN^+^GFP^+^Luc^+^ tumors and treated with M1xx, (n=12) or M28z (n=7) CAR T cells. Ten mice were included as control (n=5 injected with CD19 CAR T cells and n=5 untreated mice). (B) Comparison of the relative tumor growth (fold change from the day prior to CAR T cell treatment) between control and MSLN-CAR T cell-treated groups. (C) Kaplan-Meier curve representing the survival of the control and MSLN-CAR T cell-treated mice groups. (D) Comparison of CAR^+^ cells fraction within T cells recovered from spleen of M28z and M1xx CAR T cell-treated mice (E) CD4^+^/CD8^+^ ratio within M28z and M1xx CAR^+^ T cells isolated from mice spleen. (F) Correlation analysis of gene expression between infused M1xx and M28z CAR T cells. (G) Top 40 downregulated and upregulated genes classified by Fc (Log_2_ fold change) of expression in M1xx CAR T cells collected from mice spleen after in vivo stimulation compared with the infused CAR fraction, in blue: upregulated genes specific (ie, not found in top 60 upregulated genes in M28z) to M1xx CAR T cells. (H) Comparison of the top 40 upregulated genes between M1xx and M28z CAR T cells collected from mice spleen. Medians are represented. Two-way ANOVA with Sidak’s multiple comparison test was used to compare BLI changes between group of mice overtime. Mann-Whitney test was used to compare values between groups of mice or cell populations. Log-rank test was used to compare the survival between groups of mice. Correlation was analyzed by linear regression R^2^. *p<0.05, **p<0.01, ***p<0.001, ****p<0.0001. ANOVA, analysis of variance; BLI, bioluminescence imaging.

To compare M28z CAR constructs with a wild type or mutated CD3ζ chain, NSG mice injected i.p. with OVCAR-4 tumor cells, followed by M28z or M1xx CAR T cells i.p. injection 13 days after tumor engraftment (online supplemental figure 4A-B). Mice treated with M28z and M1xx CAR T cells presented a reduction in tumor burden as compared with control mice (five untreated and five mice injected with CD19-CAR T cells, figure 4A, B, online supplemental figure 4D). Neither M28z nor M1xx achieved tumor clearance in OVCAR-4 inoculated mice. However, tumor growth was significantly delayed in both M28z- and M1xx-treatment groups compared with the control group. Following an initial reduction in tumor burden (as determined by BLI), tumor burden reached levels comparable to initial BLI (time of CAR treatment) again+35 and +56 days post M28z or M1xx CAR T cell treatment, respectively (figure 4A). Initially, M1xx CAR T cells exerted a rapid and effective antitumor response, as shown by the up to 100-fold reduction in tumor growth 7 days post-i.p. CAR treatment. M1xx CAR T cell treatment maintained significantly lower tumor burden over time relative to the control group and M28z CAR-treated mice (p<0.001, figure 4B). Mice treated with either M28z or M1xx CAR T cells showed enhanced survival compared with control mice (p<0.001). However, M1xx CAR T cell treatment elicited superior survival compared with M28z CAR T cell-treated mice (median survival 90 days post-tumor injection vs 75 days, p<0.01, figure 4C).

T cells recovered from spleens presented higher frequencies of CAR^+^ T cells in mice treated with M1xx CAR T cells than those treated with M28z CAR T cells (p<0.01, figure 4D) both displaying predominantly an effector memory phenotype (online supplemental figure 4E). MSLN^+^ tumor cells were detected by FACS in most spleens and in mice treated with M1xx CAR T cells showed a higher CAR T cells:MSLN^+^ tumor cells ratio than M28z CAR T cell-treated mice (online supplemental figure 4F). The CD4^+^/CD8^+^ ratio was comparable between spleen-derived M28z and M1xx CAR T cells CAR^+^ M28z and M1xx T cells, however, the CD4^+^/CD8^+^ ratio was significantly higher within CAR^-^ compared with CAR^+^ fraction of M1xx-transduced T cells (p<0.05, figure 4E).

### M1xx CAR T cells exhibited a different gene expression profile than M28z CAR T cells after in vivo MSLN tumor cells stimulation

Ten CAR T cell samples were analyzed for gene expression, samples consisted of sorted CAR^+^ M28z-transduced and M1xx-transduced CAR T cells isolated from either spleen of sacrificed mice (n=8) or from the infused CAR fraction of MSLN-CAR T cells used for mice treatment (n=2) (online supplemental table 4). We first compared the gene expression profile of the infused fraction of M28z CAR T cells and M1xx CAR T cells. The profile of both infused M28z and M1xx CAR T cells was highly comparable (r^2^=0.9376, figure 4F), therefore due to their similarity, gene expression from these two infused samples were pooled together as reference when comparing to gene expression changes in spleen-derived M28z and M1xx CAR T cells.

Next, we compared the gene expression profile of MSLN-CAR T cells collected from spleens after sacrifice (52–77 days post-CAR i.p. infusion) to the gene expression profile of infused MSLN-CAR T cells. When comparing to infused CAR T cells the top 40 genes either upregulated or downregulated in M1xx and in M28z CAR T cells, most genes were common for M1xx and M28z CAR T cells (online supplemental table 5A, B). The top 40 (figure 4G, online supplemental figure 4G) most upregulated genes included dysfunction-associated genes such as *PDCD1*, *TIGIT*, *TOX*, *FOS*, *JUN* and *SLAMF6* while many of the 40 most downregulated genes were chemokine/cytokine receptors involved in Th2/Tregs profile such as *LIF*, *IL5*, *IL4*, *IL3RA*, *IL13*, *IL-9*, *CYP1B1*, *FOXP3, PTGDR2* and *IL2RA (CD25*). More specifically, *ACVR2A*, *RORC* and *CCR6;* genes involved in the Th17 differentiation profile with stem cell-like properties as well as *TCF7*, a transcription factor associated with self-renewal potential, were strongly upregulated in M1xx CAR T cells (not found in the 60 most upregulated genes in M28z CAR T cells, figure 4G, online supplemental figure 4G). *ITGA1*, encoding CD49a (expressed on repeatedly activated T cells) and *PCLAF* (highly expressed in exhausted CD8 T cells) were specifically identified within the top 40 downregulated genes in M1xx CAR T cells (figure 4G).

Lastly, we compared the gene expression profile between spleen-derived M1xx and M28z CAR T cells. M1xx CAR T cells had less upregulated genes involved in long-term activation: *IFNG, CCL4, CCL3, ITGA1* and dysfunction: *PDCD1*, *HAVCR2* (TIM-3), *NFIL3*, *TNFRSF9* and *TNSF9* (4-1BB and its ligand) compared with M28z CAR T cells but showed more gene expression associated with non-effector naïve/memory T cells such as *CCR7, CD9, BCL6 and SELL (CD62L*). Furthermore, M1xx CAR T cells differed from M28z by upregulation of *CISH* and *BCL2L1* (BCL-X) gene expression (figure 4H, online supplemental table 5C).

### Trogocytic MSLN-specific CAR T cells presented a strong activated effector phenotype ex vivo

**Figure 5:**
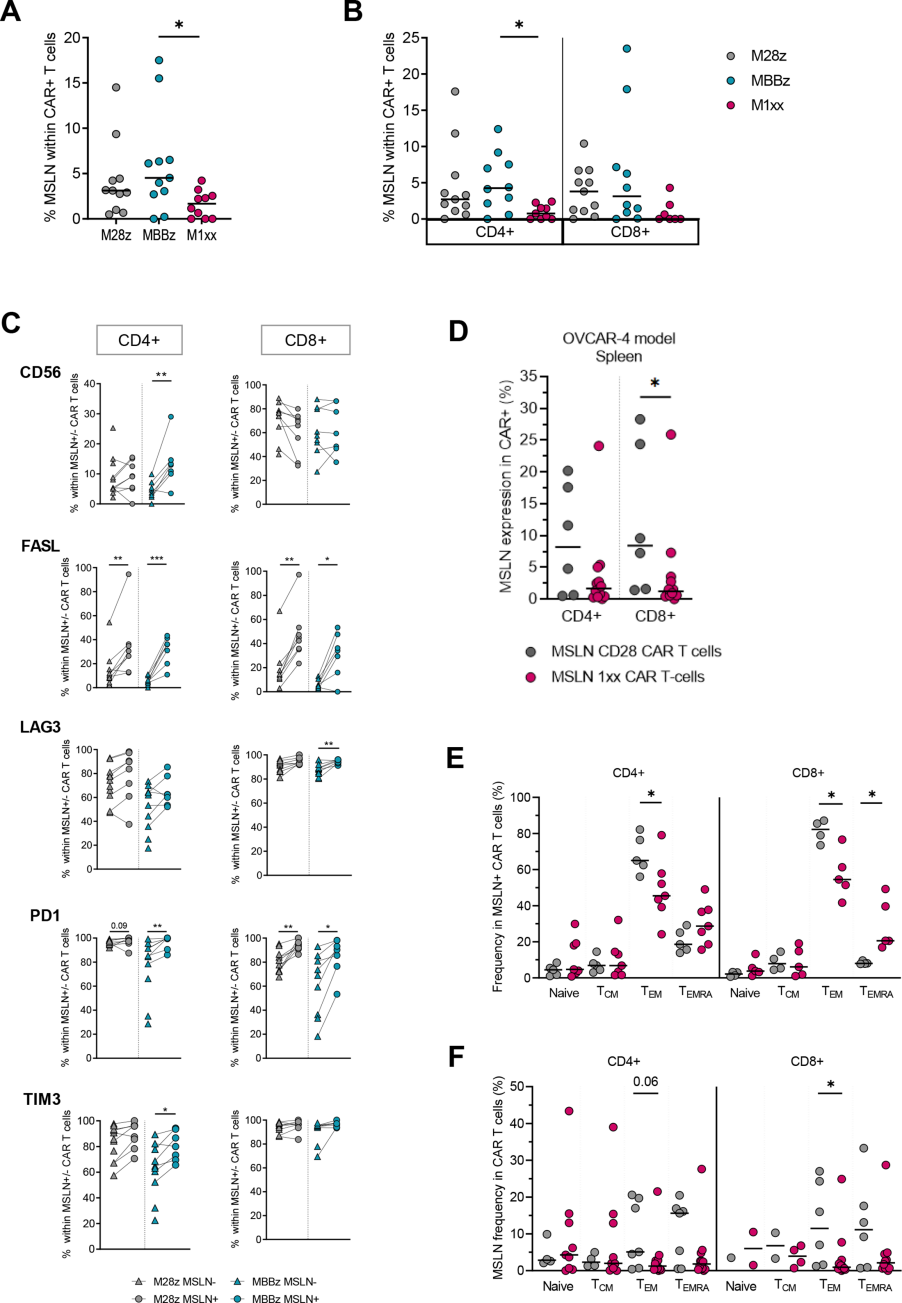
Trogocytic activity of MSLN-CAR T cells. MSLN surface expression by total CAR^+^ (A) and CD4^+^ or CD8^+^ (B) CAR^+^ MSLN-CAR T cells isolated ex vivo from SKOV-3 ovarian tumors. (C) Expression of CD56, FasL and LAG-3, PD-1 and Tim-3 in MSLN^−^ (triangles) versus trogocytic MSLN^+^ (circles) M28z and M1xx CAR T cells isolated ex vivo from primary SKOV-3 ovarian tumors. (D) detection of trogocytic MSLN^+^ cells in M28z and M1xx CAR T cells isolated from spleens of OVCAR-4 inoculated mice. (E) Comparison of the different memory subset proportions within CD4^+^ and CD8^+^ trogocytic MSLN^+^ M28z and M1xx CAR T cells derived from spleens of OVCAR-4 inoculated mice. (F) Comparison of trogocytic MSLN^+^ expression between CD4^+^ and CD8^+^ of M28z and M1xx CAR T cells within the different memory subsets. Medians are represented. Mann-Whitney and Kruskal-Wallis tests were used to compare marker expression between different 2 and 3 groups of mice, respectively. Wilcoxon matched-pairs signed rank test was used to compare marker expression between MSLN^−^ and MSLN^+^ SKOV-3 cells within same mice. *p<0.05, **p<0.01, ***p<0.001.

Since trogocytosis contributes to antigen loss and immune escape we assessed the presence of trogocytic (ie, MSLN^+^) CAR T cells. Trogocytic M28z, MBBz and M1xx CAR T cells with detectable cell surface MSLN were recovered from primary ovarian SKOV-3 tumors. We detected significantly lower frequencies of MSLN^+^ M1xx than MSLN+MBBz CAR T cells. No differences were observed between CD4^+^ and CD8^+^ MSLN-CAR T cells (figure 5A, B). FASL and PD-1 expression were elevated in MSLN^+^ (M28z and MBBz) CAR T cells compared with MSLN^-^ CAR T cells. Furthermore, CD56, LAG-3 and TIM-3 expressions were higher in MSLN^+^ MBBz CAR T cells compared with their MSLN^-^ counterparts (figure 5C).

Due to the lack of primary solid tumor samples in the OVCAR-4 mouse model, trogocytosis was assessed on spleen-derived T cells. Trogocytic MSLN^+^ M28z and M1xx CAR T cells were detected with lower levels of MSLN^+^ T cells in CD8^+^ M1xx compared with M28z CAR T cells (figure 5D). Interestingly, higher frequencies of T_EM_ were found in trogocytic MSLN^+^ M28z than M1xx CAR T cells (p<0.05) (figure 5E). Conversely, the frequency of trogocytic MSLN^+^ CAR T cells was highest within T_EM_ M28z CAR T cells relative to T_EM_ M1xx CAR T cells (p<0.05) (figure 5E, F).

## Discussion

Strategies tuning CAR activation to prevent tonic signaling and moderate signaling outputs have been developed to improve antitumor potency in both hematological and solid malignancies.^29–31^ Feucht *et al* engineered a CD19-1xx CAR construct containing mutations in the two distal ITAMs of the CD3ζ chain, which calibrated CAR activation, thereby improving functional persistence and antitumor efficacy.^20^ The promising therapeutic potential of 1xx-CAR T cells was further supported by recent studies using the tuned activated CAR T cells in the context of mesothelioma, pancreatic and melanoma mouse models.^21 22^ In the present study, we report superior tumor control by 1xx CAR T cells targeting MSLN in an ovarian solid tumor model and in a more aggressive disseminated model of ovarian cancer (high-grade serous carcinoma OVCAR-4 cell line). M1xx CAR T cells displayed a less exhausted phenotype with lower frequency of CIMs expression and superior ex vivo functional persistence and production of IFNy, TNF and GzB compared with the conventional second- generation MSLN-CAR constructs (M28z and MBBz). At the transcriptional level, this study highlighted the upregulation of genes in M1xx CAR T cells associated with naïve/memory phenotype and self-renewal potential such as *TCF7*, *CCR7*, *CD9*, *BCL6* and *SELL*^32–36^ together with the anti-apoptotic protein *BCL2L1* (BCL-X).^37^ This self-renewal gene signature is in line with the remarkable M1xx persistence observed in the long-term surviving mice and ex vivo cytokine production. Interestingly, the specific upregulation of *CISH* (an internal regulator of T cell reactivity) in M1xx CAR T cells might stem from the calibrated activation in M1xx CAR T cells. Recently, Palmer *et al*, described that *CISH* expression might inhibit expression of activation/exhaustion markers like TOX, CD39 and that PD-1 and CISH expression were mutually exclusive, suggesting that *CISH* upregulation in M1xx CAR T cells increased functional CAR T cell persistence.^38^ Superior persistence of M1xx was highlighted by the high frequency of CAR T cells recovered from the long surviving mice and detection of CAR copies in the peripheral blood. The increased frequency of CD4^+^ compared with CD8^+^ M1xx CAR T cells found within ovarian tissue of SKOV-3 inoculated mice supports the key role of CD4^+^ CAR T cells in tumor control. These findings are in line with recent findings demonstrating that two patients under long-term complete remission (>10 years) following CD19-CAR treatment presented a dominant fully functional CD4^+^ CD19-CAR T cell population.^2^ Additionally, M1xx CAR T cells upregulated Th17-related genes: *ACVR2A*, *RORC* and *CCR6*.^39–41^ The role of Th17 in tumor immunity remains debated, mediating either protumor or antitumor effect but interestingly, and in line with our observations, Th17 cells have previously been shown to harbor higher self-renewal capacity and longer in vivo survival than Th1 cells.^42–44^

We also demonstrated that M28z, MBBz and M1xx CAR T cells were capable of trogocytosis in vivo. Previous reports by our group have shown M28z-mediated and MBBz-mediated trogocytosis in in vivo and in vitro models of ovarian cancer.^27 45^ In line with previous data, trogocytic CAR T cells displayed a T_EM_ profile with higher expression levels of PD-1, LAG-3 and TIM-3, as well as CD56 and FASL, indicative of both activation and exhaustion.^45 46^ The low frequency of trogocytic M1xx CAR T cells observed in our two models suggest that superior tumor control as seen with M1xx CAR T cells correlates to lower trogocytic activity.

Following repeated antigen stimulation in vitro, M1xx CAR T cells displayed a more exhausted phenotype than M28z and MBBz CAR T cells, while in vitro killing potential was comparable between the three MSLN-CAR constructs. Importantly, CIM expression levels were reduced in ex vivo analyzed M1xx CAR T cells. We have previously demonstrated reversible CIM expression by M28z and MBBz CAR T cells following ex vivo culture in the absence of antigenic stimulation which may explain the discrepancy we observed in CIM expression by M1xx CAR T cells in vitro and ex vivo.^24^ Furthermore, repeated antigen stimulation in vitro highlighted the superior proliferative capacity and higher CCR7 expression by M1xx CAR T cells which translated into a larger frequency of T cells with a central memory phenotype. This result correlates with the gene signature found in our in vivo experiment. The increased presence of TCM M1xx CAR T cells, may therefore contribute to the improved survival following M1xx CAR T cell treatment in our study.^47 48^

While the majority of tumor-derived M28z and MBBz CAR T cells expressed CD56 which has been associated to a NK-like exhausted phenotype,^49^ the majority of tumor-derived M1xx CAR T cells lacked CD56 expression. This could be explained by the calibrated signaling in M1xx CAR T cells and/or by the fact that at time of sacrifice M1xx CAR T cells reversed to a less activated-like phenotype due to the absence/low abundance of SKOV-3 tumor cells.

Taken together, these data suggest that the reduced CIM, CD56 and MSLN expression levels by M1xx CAR T cells could be explained by the rapid M1xx-mediated clearance of tumor cells in vivo. The functional effects of trogocytosis remain to be elucidated, however, it has been shown to interfere with successful CAR-mediated antitumor responses as it promotes tumor antigen escape as well as CAR T cell dysfunction due to fratricide killing.^46^

In this study, M1xx CAR T cells elicited superior antitumor responses and outperformed conventional second-generation M28z and MBBz CAR T cells in two in vivo orthotopic models of ovarian cancer. Mutations in the two distal CD3ζ ITAMs in M1xx CAR T cells resulted in highly functional T cells, with a naïve/memory gene signature and less exhaustive phenotype, which were able to induce persistent SKOV-3 tumor control. Our study demonstrate that calibration of CAR activation can alleviate tumor-induced CAR T cells exhaustion and support the promising therapeutic potential of M1xx CAR T cells for the treatment of advanced ovarian cancer.

10.1136/jitc-2022-005691.supp10 Supplementary data

## Data Availability

Data are available on reasonable request.
