# Supplementary figures and images for: Tuned activation of MSLN-CAR T cells induces superior antitumor responses in ovarian cancer models
Source: J Immunother Cancer. 2023 Feb 1;11(2):e005691. doi: 10.1136/jitc-2022-005691 (PMC9906404; doi:10.1136/jitc-2022-005691)

Suppl. Fig. 2

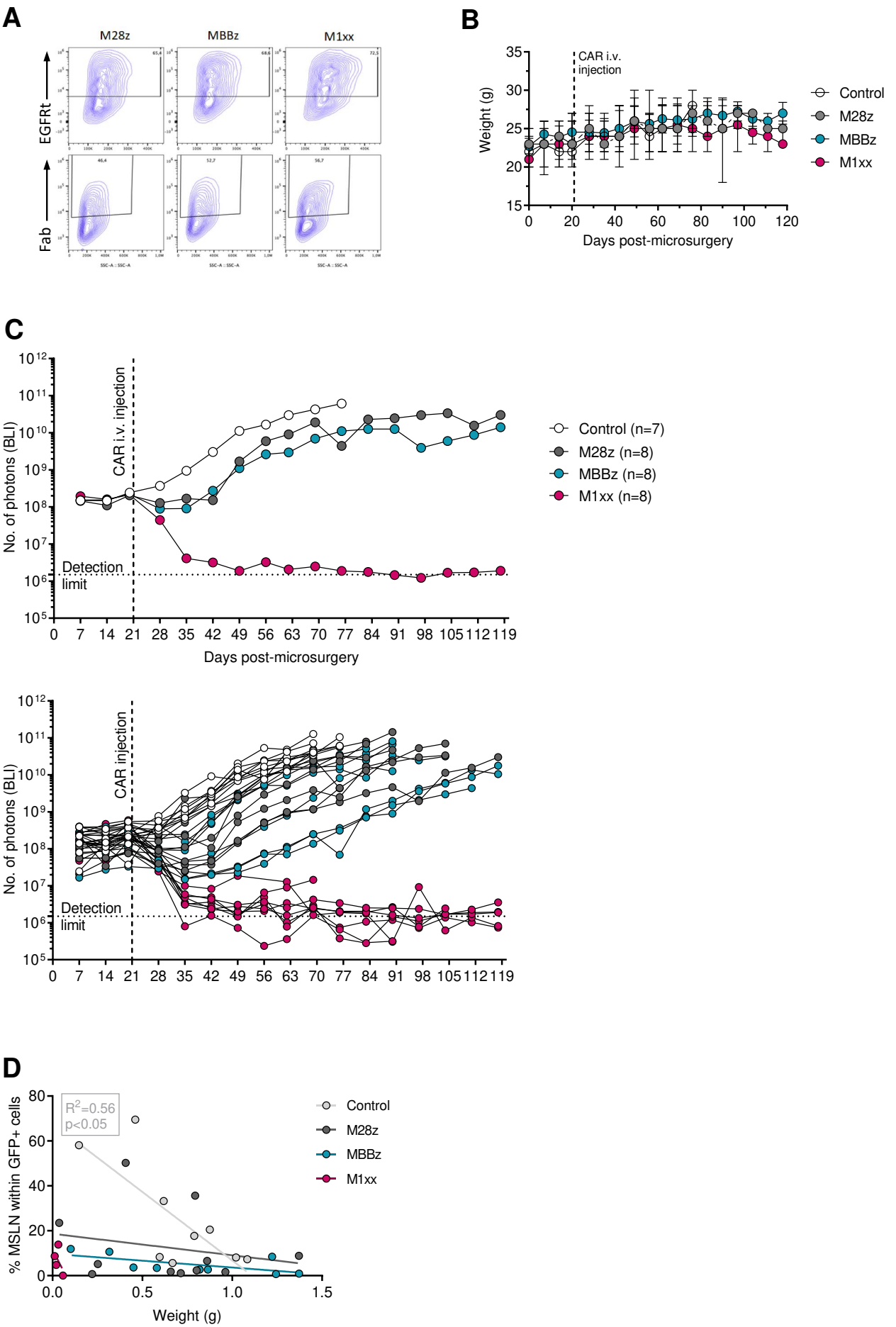

Supplement: Supplementary data [file jitc-2022-005691supp002.pdf]

Suppl. Fig. 4

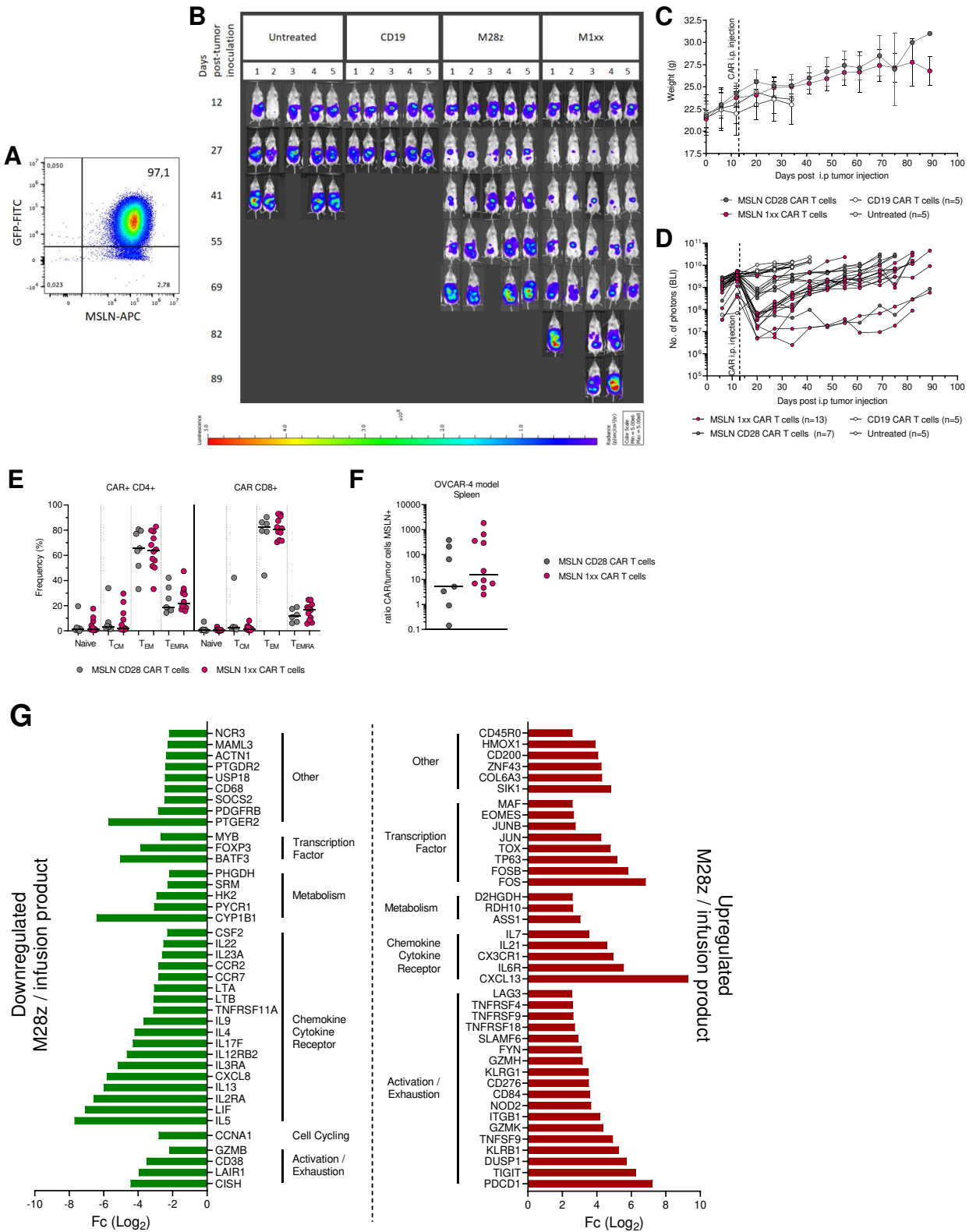

Supplement: Supplementary data [file jitc-2022-005691supp004.pdf]

Suppl. Fig. 3

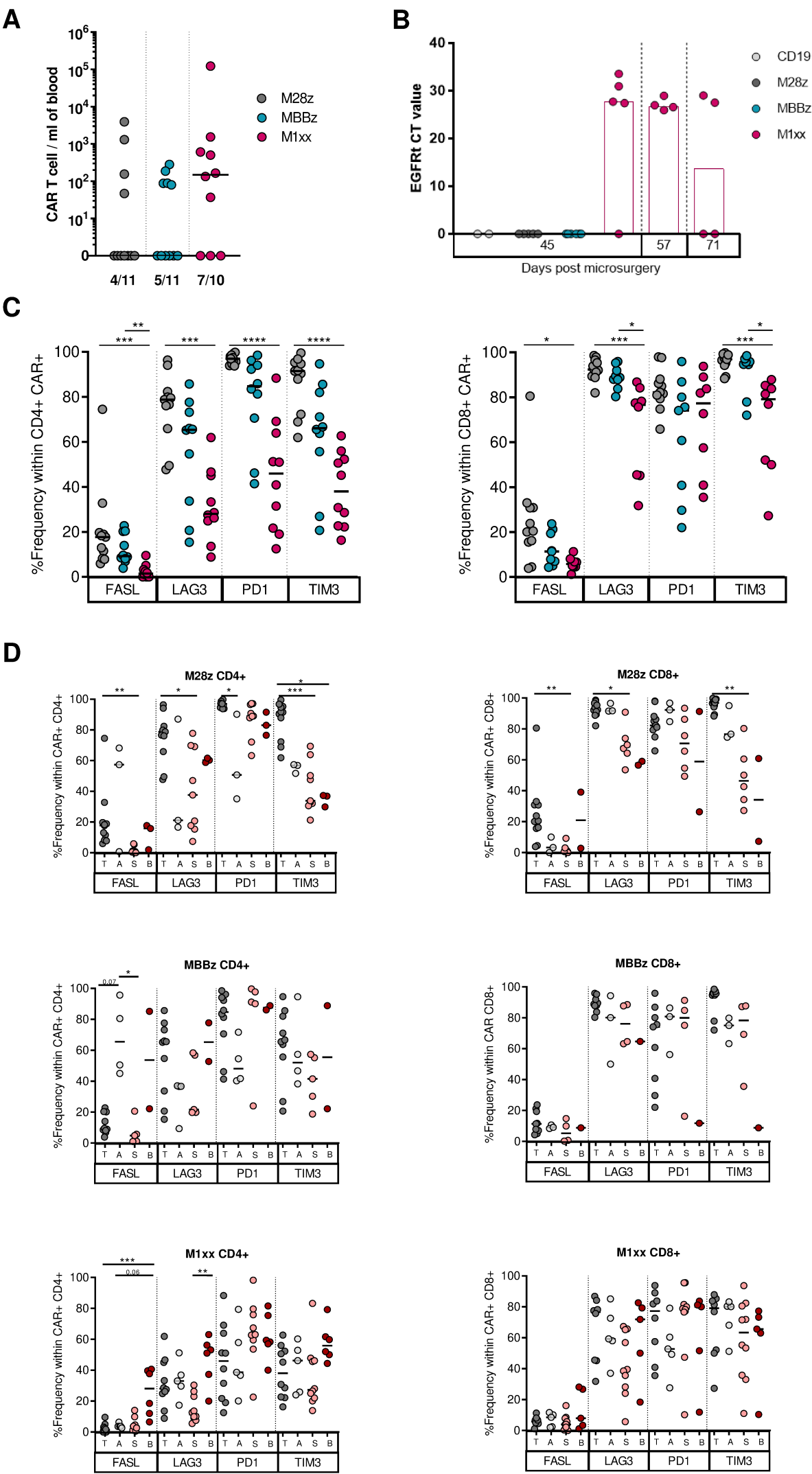

Supplement: Supplementary data [file jitc-2022-005691supp003.pdf]
