# Supplementary material for: Tuned activation of MSLN-CAR T cells induces superior antitumor responses in ovarian cancer models
Source: J Immunother Cancer. 2023 Feb 1;11(2):e005691. doi: 10.1136/jitc-2022-005691 (PMC9906404; doi:10.1136/jitc-2022-005691)
Supplement: Supplementary data [file jitc-2022-005691supp005.pdf]

Supplementary Table 1: *In vivo* study design

**SKOV-3, microsurgery**<br><br>**CAR treatment IV**,<br>Day 21-22 post-tumor	**Survival analysis**	**Intermediate sacrifice**<br>Day 62-65 post tumor	**OVCAR-4, IP**<br><br>**CAR treatment IP**,<br>Day 13 post-tumor		**Survival analysis**	**Intermediate sacrifice**<br>Day 76 post-tumor
**MBBz** (n=11)	n= 8	n=3	**M28z** (n=7)		n= 7	
**M28z** (n=11)	n= 8**	n=3	**M1xx** (n=15)		n= 13	n=2
**M1xx** (n=11)	n= 8**	n=3	**Control** (n=10)	**CD19**	n= 5	
**Control CD19** (n=9)	n= 7	n=2 +1*		**Untreated**	n= 5	

* One mouse was sacrificed due to tumor burden at the same time as two other mice sacrificed to perform analysis at intermediate timepoint. This mouse was therefore included in survival analysis. ** One mouse in M28z group and three mice in M1xx group were excluded from survival analysis because died/sacrificed for reasons unrelated to high tumor burden or its complications.
