# Supplementary material for: Tuned activation of MSLN-CAR T cells induces superior antitumor responses in ovarian cancer models
Source: J Immunother Cancer. 2023 Feb 1;11(2):e005691. doi: 10.1136/jitc-2022-005691 (PMC9906404; doi:10.1136/jitc-2022-005691)
Supplement: Supplementary data [file jitc-2022-005691supp006.pdf]

Supplementary Table 2: Flow Cytometry panels for *in vitro* assays

**Phenotype panel (*in vitro*)**	**Vendor**
EGFRt/Cetuximab (biotin-conjugated)	R&D systems
Streptavidin-PE	BioLegend®
PD-1 FITC	BD Pharmingen™
CD4-BV510	BioLegend®
CD3-AF700	BD Pharmingen™
CD8-APC-Cy7	BD Pharmingen™
LAG3-BV650	BioLegend®
TIM3-BV785/ TIM3-APC	BioLegend®
7-Aminoactinomycin D (7-AAD)	BD Pharmingen™
Cell Trace Violet	Invitrogen
**CAR T panel (*in vitro*)**	**Vendor**
EGFRt/Cetuximab (biotin-conjugated)	R&D systems
Streptavidin-PE	BioLegend®
CD3-APC	BD Pharmingen™
CD4-FITC	BD Pharmingen™
CD8-APC-Cy7	BD Pharmingen™
7-Aminoactinomycin D (7-AAD)	BD Pharmingen™
**Intracellular staining panel (*in vitro*)**	**Vendor**
CD3-PE-Cy7	BD Pharmingen™
CD4-FITC	BD Pharmingen™
CD8-APC-Cy7	BD Pharmingen™
CD107a-PE	BD Pharmingen™
TNF-eFluor610	Invitrogen
IFNy-AF647	BD Pharmingen™
IL-2-AF700	BioLegend®
**T cell phenotype (*in vitro*)**	**Vendor**
CD3-PE-Cy7	BD Pharmingen™
CD4-BV510	BioLegend®
CD8-APC-Cy7	BD Pharmingen™
CD45-AF700	BD Pharmingen™
CD45RA-BV785	BioLegend®
CCR7-BV421	BioLegend®
7-Aminoactinomycin D (7-AAD)	BD Pharmingen™
