# Supplementary material for: Tuned activation of MSLN-CAR T cells induces superior antitumor responses in ovarian cancer models
Source: J Immunother Cancer. 2023 Feb 1;11(2):e005691. doi: 10.1136/jitc-2022-005691 (PMC9906404; doi:10.1136/jitc-2022-005691)
Supplement: Supplementary data [file jitc-2022-005691supp007.pdf]

Supplementary Table 3: Flow Cytometry panels for *ex vivo* assays

**Tumor phenotype (*SKOV-3 model*)**	**Vendor**
MSLN-APC	R&D systems
CD73-PE	BD Pharmingen™
PD-L1-BV785	BioLegend®
FasL-PE-Cy7 (CD178)	BioLegend®
CX3CR1-APC-Cy7	BioLegend®
Fas-BV510 (CD95)	BioLegend®
CD3-AF700	BD Pharmingen™
7-Aminoactinomycin D (7-AAD)	BD Pharmingen™
**T cell phenotype (*SKOV-3 model*)**	**Vendor**
MSLN-APC	R&D systems
EGFRt/Cetuximab (biotin-conjugated)	R&D systems
Streptavidin-PE	BioLegend®
FasL-PE-Cy7 (CD178)	BioLegend®
PD-1-FITC	BD Pharmingen™
CD4-BV510	BioLegend®
CD3-AF700	BD Pharmingen™
CD8-APC-Cy7	BD Pharmingen™
CD56-BV421	BD Biosciences
LAG-3-BV650	BioLegend®
TIM-3-BV785	BioLegend®
7-Aminoactinomycin D (7-AAD)	BD Pharmingen™
**T cell phenotype (*OVCAR-4 model*)**	**Vendor**
MSLN-APC	R&D systems
EGFRt/Cetuximab (biotin-conjugated)	R&D systems
Streptavidin-PE	BioLegend®
CD3-PE-Cy7	BD Pharmingen™
CD4-BV510	BioLegend®
CD8-APC-Cy7	BD Pharmingen™
CD45-AF700	BD Pharmingen™
CD45RA-BV785	BioLegend®
CCR7-BV421	BioLegend®
