# Supplementary material for: Tuned activation of MSLN-CAR T cells induces superior antitumor responses in ovarian cancer models
Source: J Immunother Cancer. 2023 Feb 1;11(2):e005691. doi: 10.1136/jitc-2022-005691 (PMC9906404; doi:10.1136/jitc-2022-005691)
Supplement: Supplementary data [file jitc-2022-005691supp008.pdf]

Supplementary Table 4: List of samples for gene expression analysis

Samples ID	Mouse ID	CAR	Sacrifice days post-CAR injection
**A**		Infused M28z CAR	0
**B (pooled)**	M11	Sorted M28z	52
	M17	Sorted M28z	56
	M19	Sorted M28z	56
**C**	M14	Sorted M28z	63
**D**		Infused M1xx CAR	0
**E**	M6	Sorted M1xx	63
**F**	M7	Sorted M1xx	63
**G**	M20	Sorted M1xx	63
**H (pooled)**	M1	Sorted M1xx	70
	M5	Sorted M1xx	70
	M13	Sorted M1xx	70
**I**	M16	Sorted M1xx	77
**J (pooled)**	M2	Sorted M1xx	77
	M15	Sorted M1xx	77
