# Supplementary material for: Tuned activation of MSLN-CAR T cells induces superior antitumor responses in ovarian cancer models
Source: J Immunother Cancer. 2023 Feb 1;11(2):e005691. doi: 10.1136/jitc-2022-005691 (PMC9906404; doi:10.1136/jitc-2022-005691)
Supplement: Supplementary data [file jitc-2022-005691supp001.pdf]

Suppl. Fig. 1

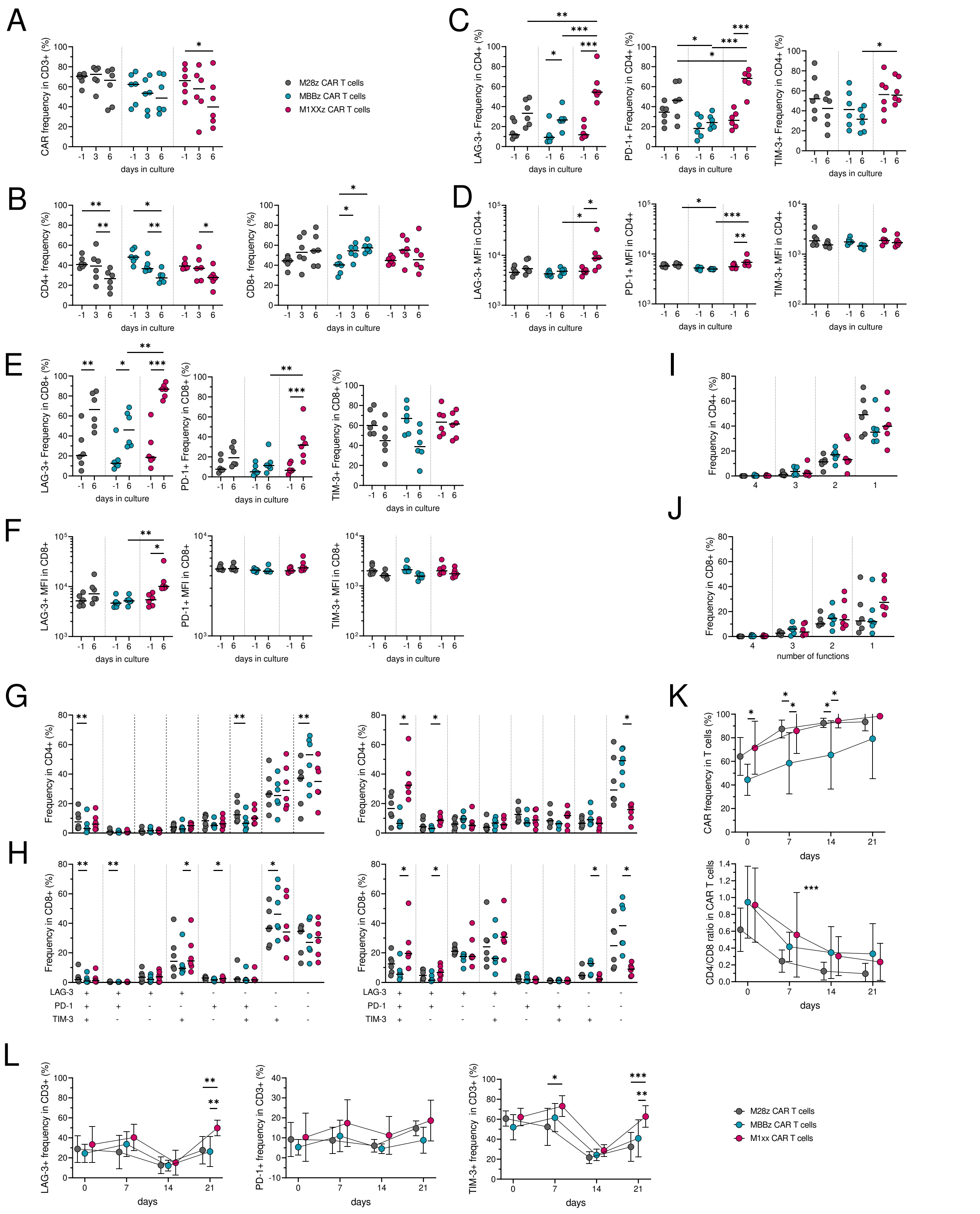
A
B
C
D
E
F
G
H
I
J
K
L
CAR frequency in CD3+ (%)
days in culture
M28z CAR T cells
MBBz CAR T cells
M1XXz CAR T cells
LAG-3+ Frequency in CD4+ (%)
PD-1+ Frequency in CD4+ (%)
TIM-3+ Frequency in CD4+ (%)
CD4+ frequency (%)
CD8+ frequency (%)
LAG-3+ MFI in CD4+
PD-1+ MFI in CD4+
TIM-3+ MFI in CD4+
LAG-3+ Frequency in CD8+ (%)
PD-1+ Frequency in CD8+ (%)
TIM-3+ Frequency in CD8+ (%)
LAG-3+ MFI in CD8+
PD-1+ MFI in CD8+
TIM-3+ MFI in CD8+
Frequency in CD4+ (%)
Frequency in CD8+ (%)
number of functions
LAG-3
PD-1
TIM-3
CAR frequency in T cells (%)
CD4/CD8 ratio in CAR T cells
days
LAG-3+ frequency in CD3+ (%)
PD-1+ frequency in CD3+ (%)
TIM-3+ frequency in CD3+ (%)
M28z CAR T cells
MBBz CAR T cells
M1xx CAR T cells
