# Supplementary material for: Tuned activation of MSLN-CAR T cells induces superior antitumor responses in ovarian cancer models
Source: J Immunother Cancer. 2023 Feb 1;11(2):e005691. doi: 10.1136/jitc-2022-005691 (PMC9906404; doi:10.1136/jitc-2022-005691)
Supplement: Supplementary data [file jitc-2022-005691supp010.pdf]

Legend Supplementary Figures 1-4 & Supplementary Table 5

Suppl. Figure 1. *In vitro* stimulation of MSLN-CAR T cells. A-J. Short term restimulations assay. **A.** Frequency of CAR^+ cells within M28z, MBBz and M1xx-transduced T cells. **B.** $CD4^+$ and $CD8^+$ frequency in MSLN-CAR T cells. **C-F.** Comparison of LAG-3, PD-1, and TIM-3 CIM expression (**C** and **E**) and MFI (**D** and **F**) in $CD4^+$ (**C-D**) and $CD8^+$ (**E-F**) CAR T cells between M28z, MBBz and M1xx CAR T cells prior to and after multiple antigenic stimulations. **G-H.** Comparison between different MSLN-CAR $CD4^+$ (**G**) and $CD8^+$ (**H**) T cells of immune checkpoint markers co-expression of before (left) and after (right) multiple stimulation. **I-J.** Frequency of CD107a, IFNγ, IL-2 and TNF producing $CD4^+$ (**I**) and $CD8^+$ (**J**) T cells by multifunctionality after multiple stimulations. **K-L.** Long term restimulations assay **K.** CAR frequency and $CD4^+/CD8^+$ ratio of the different MSLN-CAR T cells over the period of 3 weeks with weekly antigenic stimulations. **L.** LAG-3 (left), PD-1 (middle), and TIM-3 (right) expressions in CAR T cells between M28z, MBBz and M1xx CAR T cells prior to and after 3 antigenic stimulations over the period of 3 weeks. The 2-way ANOVA with Sidak's test was used to do multiple comparisons between CAR T cells and date. Friedman test with Dunn's correction was used to compare the 3 paired MSLN-CAR T cells. All assay were performed using 6 healthy donors, N=6. Delta Δ= value at d6 – value at d0. Medians are represented. *$p<0.05$, **$p<0.01$, ***$p<0.001$.

Suppl. Figure 2. Monitoring the SKOV-3 ovarian cancer animal model. A. Representative plot of anti-EGFRt (top) and anti-human Fab (anti-CAR) staining of the different infused MSLN-CAR T cells. **B.** Weekly monitoring of the NSG mice weight injected with SKOV-3 $MSLN^+GFP^+Luc^+$ tumors and treated with MSLN 1xx CAR T cells, MSLN CD28 CAR T cells or control (CD19 CAR T cells). **C.** Median (top) and individual (bottom) BLI weekly monitoring of the different NSG mice groups. Detection limit of lowest BLI (1.5E6 photons) is represented by horizontal dotted line. **D.** Reverse correlation between MSLN expression in GFP^+ SKOV-3 cells and tumor weight between the different groups of mice.

Suppl. Figure 3. *Ex vivo* characterization of MSLN-specific CAR T cells. A. Normalized CAR T cell count per ml of blood recovered at sacrifice (heart puncture). **B.** EGFRt CT values in blood (tail vein) overtime. **C.** Comparison of FasL, LAG-3, PD-1 and TIM-3 expression in $CD4^+$ and

$CD8^+$ CAR T cells isolated from *ex vivo* tumors between M28z, MBBz and M1xx CAR T cells groups. **D**. Comparison of FasL, LAG-3, PD-1 and TIM-3 expression in $CD4^+$ and $CD8^+$ CAR T cells between organs (tumor vs. ascites vs. spleen vs. blood) in the different mice treatment groups. Kruskal-Wallis test was used to compare the antigen expression between the different 3 groups of mice. Medians are represented. *p<0.05, **p<0.01, ***p<0.001, ****p<0.0001.

Suppl. Figure 4. Monitoring of disseminated OVCAR-4 animal model and *ex vivo* MSLN-CAR T cells characterization. A. Representative plot of MSLN expression in OVCAR-4 ovarian cancer cells. **B**. Representative weekly bioluminescence monitoring of NSG mice injected with OVCAR-4 $MSLN^+GFP^+Luc^+$ tumors and treated with M1xx CAR T cells, M28z CAR T cells or control (untreated and control treatment with CD19-CAR T cells). **C.** Weekly monitoring of NSG mice weight. **D.** Overall individual graphical representation of the intraperitoneal OVCAR-4 tumor burden of NSG mice. **E.** Memory phenotype, as determined by CD45RA and CCR7 expression, of $CD4^+$ and $CD8^+$ of M28z or M1xx CAR T cells recovered from spleen of mice at sacrifice. **F.** Ratio of CAR+ T cells: MSLN+ tumor cells in spleen of M28z- or M1xx-treated mice. **G.** Top 40 downregulated and upregulated genes classified by Fc (Log_2 Fold change) of expression of the M28z CAR T cells collected from mice spleen after *in vivo* stimulation in comparison to the infused CAR fraction.

Supplementary Table 5 (Available as Excel document): A. List of the genes used for the Fold Changes (Log_2) between M1xx CAR T cells collected from sacrificed mice and the original infused CAR T cell fraction. **B.** List of the genes used for the Fold Changes (Log_2) between M28z CAR T cells collected from sacrificed mice and the original infused CAR T cell fraction. **C.** List of the genes used for the Fold Changes (Log_2) between M28z CAR T cells and M1xx CAR T cells collected from sacrificed mice.
